# The Potential Role of *PeMAP65-18* in Secondary Cell Wall Formation in Moso Bamboo

**DOI:** 10.3390/plants13213000

**Published:** 2024-10-27

**Authors:** Yuhan Jia, Shuxin Chen, Mengyun Li, Longfei Ouyang, Jing Xu, Xiaojiao Han, Wenmin Qiu, Zhuchou Lu, Renying Zhuo, Guirong Qiao

**Affiliations:** 1 State Key Laboratory of Tree Genetics and Breeding, Zhejiang Key Laboratory of Forest Genetics and Breeding, Research Institute of Subtropical Forestry, Chinese Academy of Forestry, Hangzhou 311400, China; jiayuhan022223@caf.ac.cn (Y.J.); chenshuxin317@caf.ac.cn (S.C.); limengyun@caf.ac.cn (M.L.); longf5555@caf.ac.cn (L.O.); cafxujing@caf.ac.cn (J.X.); hanxj@caf.ac.cn (X.H.); qiuwm05@caf.ac.cn (W.Q.); luzc@caf.ac.cn (Z.L.); zhuory@caf.ac.cn (R.Z.); 2 College of Landscape Architecture, Nanjing Forestry University, Nanjing 210037, China

**Keywords:** *Phyllostachys edulis*, microtubule-associated proteins, secondary cell walls, cellulose, gene family

## Abstract

Microtubule-associated proteins (MAPs) play a pivotal role in the assembly and stabilization of microtubules, which are essential for plant cell growth, development, and morphogenesis. A class of plant-specific MAPs, MAP65, plays largely unexplored roles in moso bamboo (*Phyllostachys edulis*). This study identified 19 *PeMAP65* genes in moso bamboo, systematically examining their phylogenetic relationships, conserved motifs, gene structures, collinearity, and *cis*-acting elements. Analysis of gene expression indicated that *PeMAP65*s exhibit tissue-specific expression patterns. Functional differentiation was investigated among the members of different *PeMAP65* subfamilies according to their expression patterns in different development stages of bamboo shoots. The expression of *PeMAP65-18* was positively correlated with the expression of genes involved in secondary cell wall (SCW) biosynthesis. Y1H and Dual-LUC assays demonstrated that the transcription of *PeMAP65-18* was upregulated by PeMYB46, a key transcription factor of SCW biosynthesis. The result of subcellular localization showed that PeMAP65-18 was located in cortical microtubules. We speculate that *PeMAP65-18* may play a crucial role in the SCW deposition of moso bamboo. This comprehensive analysis of the MAP65 family offers novel insights into the roles of *PeMAP65*s in moso bamboo, particularly in relation to the formation of SCWs.

## 1. Introduction

Bamboo, a perennial herbaceous plant, is widely distributed across temperate and tropical forests, characterized by lignified secondary cell walls (SCWs) similar to those of woody trees. Moso bamboo (*Phyllostachys edulis*), in particular, dominates nearly 73% of China’s bamboo forests, covering approximately 6.4 million hectares [1]. As a fast-growing species, moso bamboo reaches maturity within about four months, with its hollow internodal structure and layered cell walls conferring excellent mechanical properties [2]. It is highly valued in industries such as construction, papermaking, and furniture owing to its strong fibers and unique material properties [3]. The cell wall, as the primary load-bearing component of bamboo fiber cells, features a unique structure comprising primary walls and alternating thin–thick multilayered secondary walls. This configuration imparts high rigidity, straight morphology, and stable performance, resulting in exceptional physical and mechanical properties [4]. Given the growing demand for non-timber resources due to wood scarcity and energy depletion, understanding bamboo’s developmental processes, especially the regulation of SCW formation, is essential for enhancing its industrial applications.

The process of cell wall formation involves not only the synthesis of macromolecules but also the transport of their precursors to the deposition site, where they are eventually integrated and assembled [5]. Microtubules (MTs) and microfilaments within the cytoskeleton regulate the spatiotemporal transport and deposition of cell wall materials, thereby promoting overall cell wall formation in plant cells [6,7]. Organized into parallel arrays along the plasma membrane, microtubules control the pattern of cellulose deposition. Numerous MAPs function as linkers between cellulose synthase complexes (CSCs) and microtubules, playing a pivotal role in the early stages of cellulose synthesis [8]. In recent years, with the identification and functional research of cortical microtubule-associated proteins, the mechanism by which cortical microtubules mediate the thickening pattern of SCWs has been partially elucidated [9]. As one of the important components of the cytoskeleton, MTs play an extremely important role in eukaryotic life. In many physiological activities, MTs regulate their own dynamic changes and the array of different cell stages by interacting with MAPs, and they participate in maintaining cell morphology and structure, constructing cell walls and regulating cytoplasmic flow, polar cell growth, cell mitosis, cell differentiation, and intracellular signal transduction [10,11]. Therefore, the regulatory mechanism of MAPs on MTs has been widely investigated in plant biology.

At present, the MAPs discovered in plants have been divided into 12 families, including SPR1, WVD2, and MAP65 [12]. Among them, MAP65 has the most members and is a plant-specific MAP [13]. MAP65 proteins are typically localized to one or more microtubule arrays, where they act as microtubule-binding proteins that cross-link with microtubules and promote the formation of microtubule bundles [14]. MAP65s were first discovered in tobacco (*Nicotiana tabacum*) [15]. *Arabidopsis thaliana* contains nine MAP65 family members. [16]. In addition to tobacco and *Arabidopsis*, nine and eleven members of MAP65s were identified in *Populus trichocarpa* and *Oryza sativa*, respectively [17]. Five *MAP65*s were cloned in *Physcomitrella patens* [18]. *MAP65*s were also reported in carrot (*Daucus carota*) [19], zinnia (*Zinnia elegans*) [20], cowpea (*Vigna sinensis*) [21], maize (*Zea mays*) [22], and grapevine (*Vitis vinifera*) [23]. Early studies on MAP65s mainly focused on in vitro experiments to analyze how to regulate the polymerization, assembly, alignment, and stability of microtubules [24]. Phenotypic analysis has also been reported, and relationships have been established between microtubules and plant resistance [25], axial root growth, cytokinesis [26], embryo sac development [27], and so on. However, few studies have reported on how microtubule-associated proteins participate in the formation of the SCW.

In this study, MAP65 proteins were identified on a genome-wide scale in moso bamboo. Phylogenetic relationships, conserved motifs, intron-exon distribution, collinearity, and *cis-*acting elements were investigated. The expression profiles of *PeMAP65*s across varying tissues and developmental stages were scrutinized. Co-expression and hierarchical gene regulatory networks were constructed. The upstream transcription factors that may regulate *PeMAP65*s were also assessed, and their regulatory relationship was experimentally verified. Further investigation on the potential role of PeMAP65-18 in microtubule bundling was performed by analyzing its subcellular localization. The results offer an all-encompassing assessment of the *MAP65* gene family in moso bamboo, thereby enriching our comprehension of the role of *MAP65*s in SCW formation.

## 2. Results

### 2.1. Identification of the MAP65s in Moso Bamboo

In this study, 19 *PeMAP65*s were identified and designated *PeMAP65-1* through *PeMAP65-19* based on their chromosomal positions (Table 1). The molecular weights of PeMAP65 proteins varied, with PeMAP65-13 having the smallest size at 510 amino acids and PeMAP65-10 the largest at 687 amino acids. Additionally, the relative molecular masses varied between 58.70 kDa (PeMAP65-13) and 78.07 kDa (PeMAP65-10). The theoretical isoelectric points (pI) for the PeMAP65 proteins spanned from 5.07 (PeMAP65-6) to 8.2 (PeMAP65-12). All PeMAP65 proteins were unstable proteins (stability coefficient ≥ 40) with stability coefficients ranging from 43.80 (PeMAP65-11) to 63.80 (PeMAP65-13). The subcellular localization prediction indicated that eleven PeMAP65 proteins are located in the cytoplasm, five in the nucleus, two in the chloroplast, and one in the endoplasmic reticulum.

### 2.2. Phylogenetic Analysis of PeMAP65s

**Figure 1:**
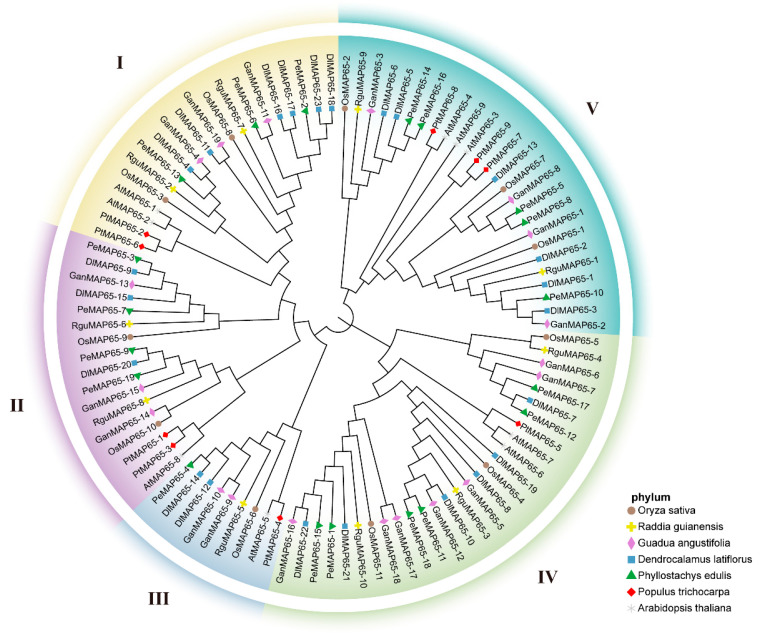
Phylogenetic tree of MAP65 gene family in seven species. Phylogenetic analysis of MAP65s across *Phyllostachys edulis*, *Raddia guianensis*, *Dendrocalamus latiflorus*, *Guadua angustifolia*, *Oryza sativa*, *Populus trichocarpa*, and *Arabidopsis thaliana*. Roman numerals I, II, III, IV, and V denote distinct gene clusters (groups). Variations in symbol shapes and colors represent different species.

A phylogenetic analysis was performed to investigate the relationship of MAP65s in moso bamboo and other plant species, including three model plants (*A. thaliana*, *O. sativa*, and *P. trichocarpa*) and three other bamboo species (*D. latiflorous*, *G. angustifolia*, and *R. guianensis*) (Figure 1). The phylogenetic tree was divided into five subclades, with MAP65s from these species present in nearly all branches, suggesting the conservation of this gene family across different species throughout evolution. A total of 100 genes were classified into five distinct subfamilies, with the subfamily IV containing the largest number of members, amounting to 28. This subfamily comprised two AtMAP65s, two OsMAP65s, one PtMAP65, six PeMAP65s, three RguMAP65s, seven GanMAP65s, and six DlMAP65s. The subfamily V followed with 26 members, comprising 3 AtMAP65s, 3 OsMAP65s, 3 PtMAP65s, 5 PeMAP65s, 2 RguMAP65s, 4 GanMAP65s, and 6 DlMAP65s. The subfamily III contained the fewest genes, with only nine members. In this subfamily, two tropical woody bamboos, *D. latiflorus* and *G. angustifolia*, each had two members, while the other species possessed only a single member. In subfamily I, both PeMAP65s and GanMAP65s had an equal number of members, with three each, while *D. latiflorus* had six members, and *R. guianensis* contained only two members.

### 2.3. Analysis of Gene Structure and Conserved Motifs

**Figure 2:**
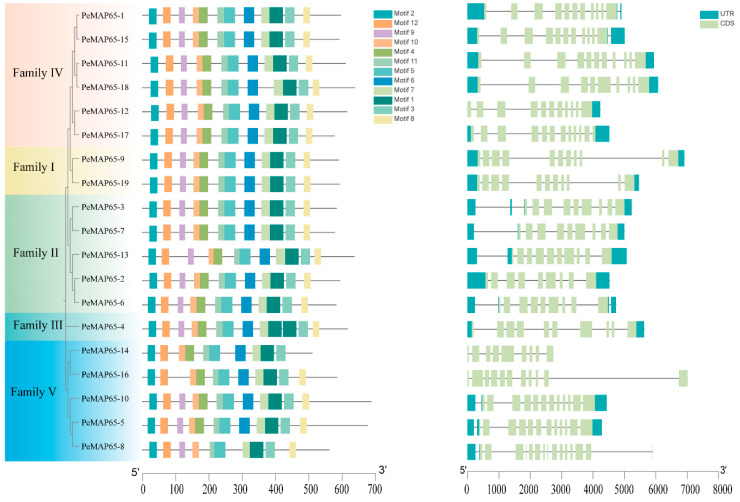
Conserved motifs and gene structure of PeMAP65s. The motif distribution of MAP65s (**left**). A total of 12 motifs are represented, each within differently colored boxes. Gene structures of *PeMAP65*s (**right**). The light green box represents the exon, the blue-green box represents the UTR, and the black line indicates the intron.

To further clarify the characteristics of the MAP65 family in moso bamboo, 12 conserved motifs were identified across 19 PeMAP65s (Figure 2). In subfamilies I, II, and IV, PeMAP65s contained these 12 motifs. One repetition of motif1 occurred in PeMAP65-4, which belonged to subfamily III. In subfamily V, motif8 and motif9 were absent in PeMAP65-14, motif6 was absent in PeMAP65-8, and motif9 was absent in PeMAP65-16. The genomic DNA sequence lengths of *PeMAP65*s varied from 2735 bp (*PeMAP65-14*) to 7016 bp (*PeMAP65-16*). *PeMAP65*s contained 9 to 15 exons (Figure 2). *PeMAP65*s grouped within the same subfamily exhibited similar gene structures; for instance, the two members in subfamily I contained 11 exons, while members in subfamily II typically had 9–10 exons, and the number of exons in subfamily V ranged from 9 to 15.

### 2.4. Analysis of Cis-Acting Elements in the Promoters of PeMAP65s

**Figure 3:**
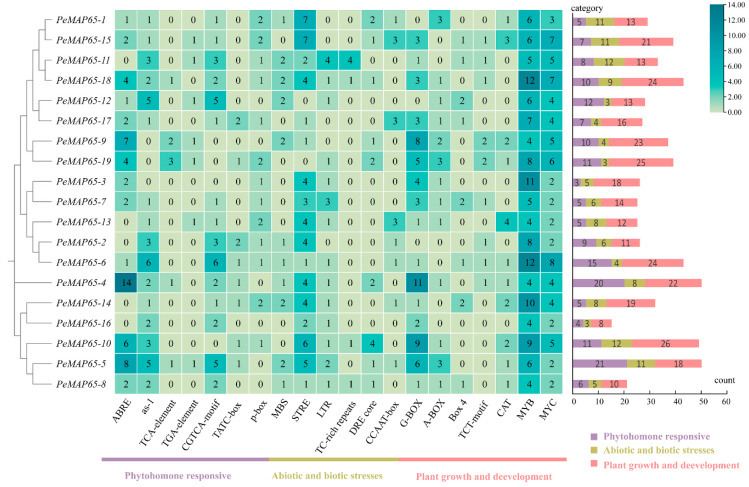
*Cis*-acting elements predicted in the promoter of *PeMAP65*s. All *cis*-acting elements were categorized into three categories. The heatmap demonstrates the number of *cis*-acting elements with the higher number in blue and the lower number in yellow. On the right, the number in the differently colored boxes represents the count of *cis*-acting elements in each category.

As shown in Figure 3, the promoters of *PeMAP65*s contain a wide variety of *cis*-acting elements involved in phytohormone responses, abiotic and biotic stresses, and plant growth and development. Four *PeMAP65*s contain more than five ABRE elements that respond to abscisic acid (ABA). Stress-responsive elements (STREs) are distributed in the promoter regions of 17 *PeMAP65*s. MYB and MYC elements related to development and cell cycle regulation were found in all *PeMAP65*s. Four *PeMAP65*s, *PeMAP65-3*, *PeMAP65-4*, *PeMAP65-14*, and *PeMAP65-18*, contain 10 or more MYB elements. These findings suggest that *PeMAP65*s may exert their biological functions through various signaling pathways.

### 2.5. Chromosomal Location, Gene Duplication, and Synteny Analysis

**Figure 4:**
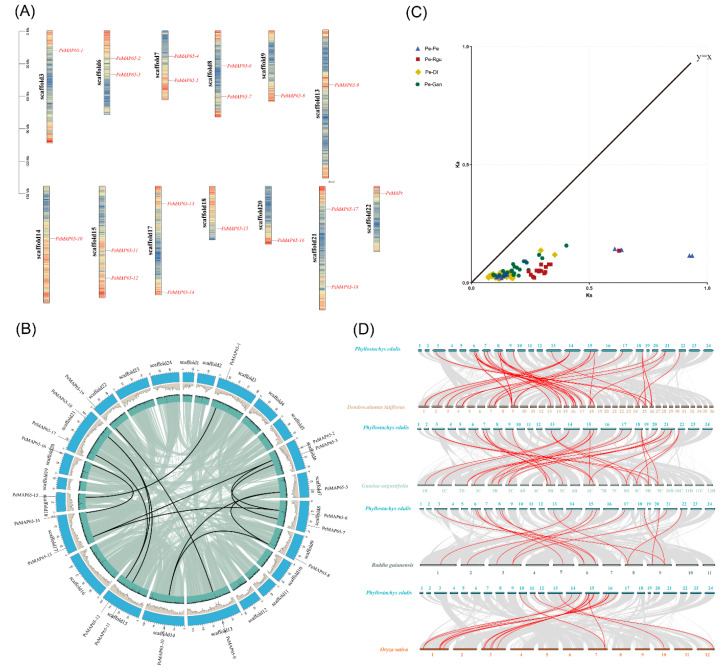
Chromosomal location and synteny analysis of *PeMAP65*s. (**A**) The distribution of the *PeMAP65*s located on the chromosomes in *P. edulis*. The genetic distance of 13 chromosomes are represented by the scale in megabases (Mb) on the left. Black lines represent the location of the gene on each chromosome. (**B**) Collinearity analysis of the *PeMAP65* gene family. Each *PeMAP65* is marked with a short black line on the chromosome, and collinear gene pairs are represented by a black curve. (**C**) The *Ka* (non-synonymous substitution) and *Ks* (synonymous substitution) values of *MAP65*s were calculated. Pe, *P. edulis*, Rgu, *R. guianensis*, Dl, *D. latiflorus*, Gan, *G. angustifolia.* (**D**) Collinearity analysis of *MAP65*s between *P. edulis* and three other bamboo species and rice. The gray lines indicate collinear blocks within the *P. edulis* genome and other plant genomes, and the red curves indicate *MAP65*s with collinearity.

These 19 *PeMAP65*s were distributed among 13 chromosomes, each containing one or two *PeMAP65*s, and no tandem repeat events were found (Figure 4A). The evolutionary time of homologous gene pairs was calculated based on *Ks* values (Table S2). The earliest gene duplication of *PeMAP65* occurred around 51.43 million years ago, and the most recent around 5.86 million years ago. The divergence time for *MAP65*s between *P. edulis* and *R. guianensis* was estimated to be between 34.40 million years (*PeMAP65-16/RguMAP65-9*) and 13.31 million years (*PeMAP65-9/RguMAP65-8*) ago. Between *P. edulis* and *G. angustifolia*, divergence ranges from 22.11 million years (*PeMAP65-16/GanMAP65-3*) to 5.09 million years (*PeMAP65-9/GanMAP65-15*). For *D. latiflorus*, divergence ranges from 19.43 million years (*PeMAP65-16/DlMAP65-8*) to 3.97 million years (*PeMAP65-9/DlMAP65-42*). The *Ka*/*Ks* ratios between *P. edulis* and all other bamboo species were less than one, indicating that the *MAP65* gene family has undergone purifying selection during its evolution (Figure 4B). Except for *PeMAP65-4*, a total of 13 putative paralogous gene pairs were identified among 18 *PeMAP65*s by collinearity analysis in *P. edulis* (Figure 4B). The analysis of the collinearity relationships among *MAP65*s in *P. edulis*, *R. guianensis*, *G. angustifolia*, and *D. latiflorus* revealed that all bamboo species possessed corresponding orthologous genes. The results showed that *P. edulis* shared 15 homologous gene pairs with *R. guianensis*, 25 with *G. angustifolia*, and 53 with *D. latiflorus* (Figure 4C). The *MAP65* gene family was relatively conserved and structurally stable across these bamboo species, suggesting similar functional roles.

### 2.6. Expression Patterns of PeMAP65s

**Figure 5:**
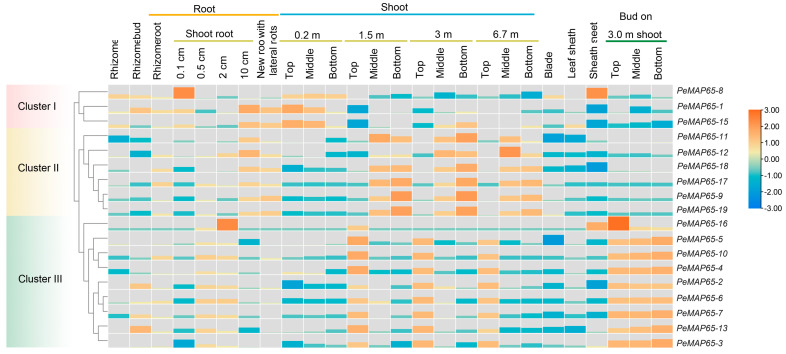
The expression patterns of *PeMAP65*s in 26 tissues. The heatmap represents the expression levels of *PeMAP65*s calculated by Log2FPKM.

The expression patterns of *PeMAP65*s in 26 diverse tissues and developmental phases were analyzed according to RNA-seq data from moso bamboo (Figure 5). Except for *PeMAP65-14*, which was not expressed in any tissue, most *PeMAP65*s exhibited tissue specificity in their expression and were classified to three clusters. In cluster I, the expression levels of *PeMAP65-1*, *PeMAP65-8*, and *PeMAP65-15* in the shoots at 0.2 m height were higher than those at greater heights. In cluster II, *PeMAP65*s were highly expressed in the middle and lower parts of the shoot, while *PeMAP65*s in cluster III were highly expressed in the upper parts of shoots and shoot buds.

**Figure 6:**
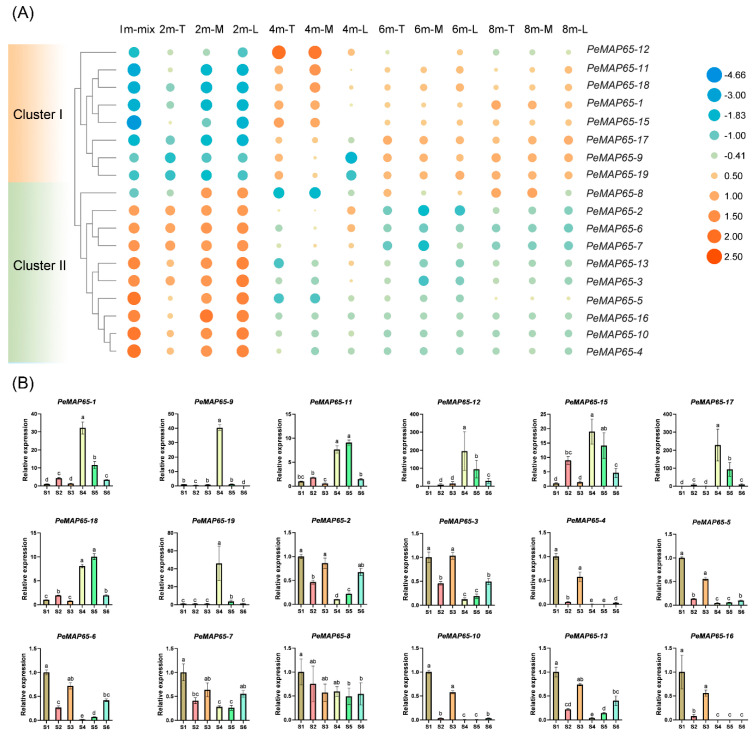
The expression pattern of *PeMAP65*s in bamboo shoots with different degrees of lignification. (**A**) The heatmap represents the expression level (Log2FPKM) of *PeMAP65*s at the 13th internode of bamboo shoots with different heights. T, M, and L represent the top, middle, and lower portions of the 13th internode. (**B**) The relative expression levels of *PeMAP65*s in different internodes of moso bamboo shoots with a height of 2.5 m. S1 represents the 22nd internode. S2 and S3 represent the top and lower portions of the 18th internode. S4 and S6 represent the top, middle, and lower portions of the 13th internode. Data represent means (±SD) of three biological replicates. The different letters indicate significant differences, for which the relative expression level between two samples was greater than or equal to 2.

It could be found that *PeMAP65*s may be related to the growth and lignification process of bamboo shoots. Consequently, the expression of *PeMAP65*s was further characterized by investigating their expression profiles within the 13th internode of shoots at different heights. It can be clearly observed that the expression of *PeMAP65*s was mainly divided into two clusters (Figure 6A). Cluster I contained eight *PeMAP65*s, whose expression was upregulated along with the lignification process of bamboo shoots. However, the 10 *PeMAP65*s in cluster II showed opposite expression trends, mainly being highly expressed in 1 m and 2 m bamboo shoots. These results indicated that different *PeMAP65* members played different roles in different developmental stages of bamboo shoots. The relative expression of *PeMAP65*s were validated by qRT-PCR (Figure 6B). During rapid bamboo growth, the degree of lignification varies across different internodes within the same bamboo shoot, as well as within different sections of the same internode. The samples used in qRT-PCR were ranked in terms of lignification degree from low to high, denoted as S1, S3, S2, S6, S5, and S4. The results revealed that these *PeMAP65*s exhibited similar expression patterns consistent with the findings of the transcriptome analysis.

### 2.7. Co-Expression Network

**Figure 7:**
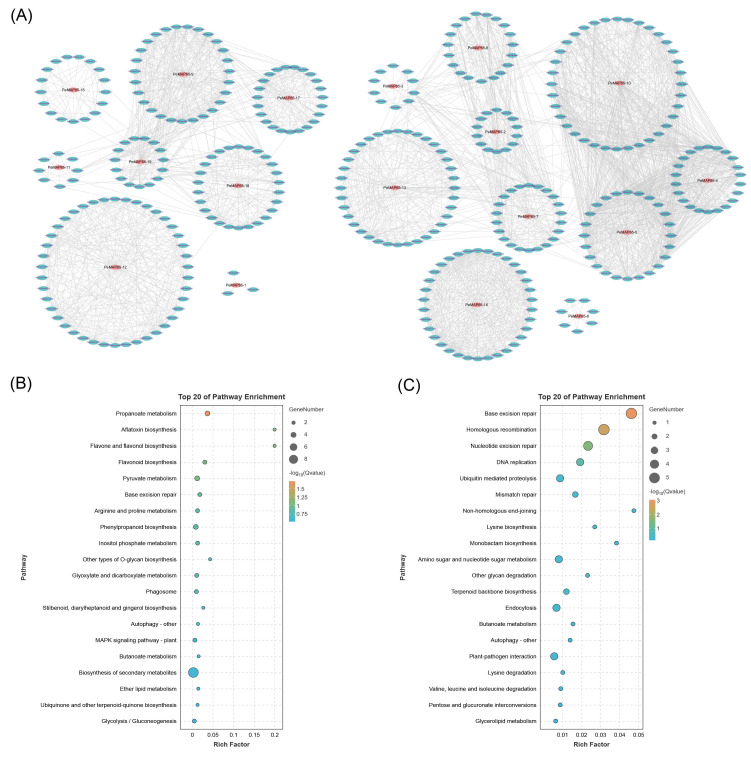
Co-expression networks of PeMAP65s. (**A**) Co-expression networks constructed by the database of co-expression networks with functional modules for moso bamboo. Red ellipses represent *PeMAP65*s, green ellipses represent the co-expressed genes with *PeMAP65*s, and gray lines represent the edges between these co-expressed genes. (**B**,**C**) KEGG functional enrichment on the genes co-expressed with *PeMAP65*s in two clusters based on the expression patterns of *PeMAP65*s in different lignified shoots.

Using *PeMAP65*s as the node genes, a co-expression network was constructed based on the database of co-expression networks with functional modules for moso bamboo. A total of 1297 genes exhibited co-expression with 18 *PeMAP65*s, resulting in the sharing of 2872 edges (Figure 7A). Based on the expression patterns of *PeMAP65*s in different lignified shoots, we conducted KEGG functional enrichment analysis on the genes co-expressed with *PeMAP65*s in two clusters. The KEGG functional enrichment results indicate that the genes in cluster I were mainly enriched in biosynthesis of secondary metabolites (Figure 7B), with significant enrichment in phenylpropanoid biosynthesis, while the genes in cluster II were mainly enriched in areas such as base excision repair, homologous recombination, DNA replication, and so on (Figure 7C). These results indicated that *PeMAP65*s in these two clusters played different functions in bamboo growth and development.

### 2.8. The Transcriptional Level of MAP65-18 Was Upregulated by PeMYB46

**Figure 8:**
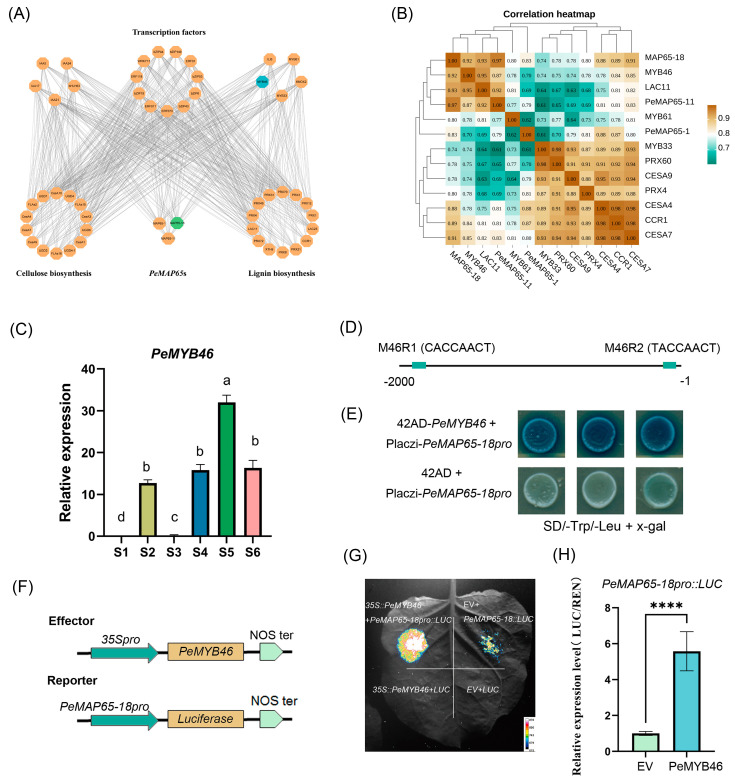
The transcriptional level of *PeMAP65-18* was upregulated by PeMYB46. (**A**) A hierarchical gene regulatory network. At the top level, *MYB46* (blue) is shown as a direct target of *MAP65-18* (green). (**B**) The correlation heatmap of *PeMAP65*s with the genes related to SCW biosynthesis. The data represent the Pearson coefficient. (**C**) The relative expression level of *PeMYB46* in different internodes. The different letters indicated significant differences, which the relative expression level between two samples was greater than or equal to 2. (**D**) Schematic diagram of MYB binding sites on the *PeMAP65-18* promoter. (**E**) Y1H validation of the interaction between PeMYB46 and *PeMAP65-18* promoter. (**F**) Schematic diagrams of the effector (*PeMYB46*) and reporter (*PeMAP65-18*pro) constructs were used in the dual-luciferase reporter assay. (**G**) An image of luciferase activity. (**H**) Relative luciferase activity was measured. Error bars represent SD. Statistical analysis was performed using the *t* test (n = 7, ****, *p* < 0.0001).

Utilizing a bottom-up Gaussian graphical model (GGM) algorithm, a hierarchical regulatory network was constructed from transcriptomic data (Figure 8A). Based on the hierarchical regulatory network, three *PeMAP65*s (*PeMAP65-1*, *PeMAP65-11*, and *PeMAP65-18*) were co-expressed with numerous genes involved in lignin and cellulose biosynthesis. Based on the expression levels within the 13th internode of shoots at different heights, ten genes including three transcription factors (*MYB33*, *MYB46*, and *MYB61*), four phenylpropanoid biosynthesis pathway genes (*CCR1* and *PRX43*, *PRX60* and *LAC11*), and three cellulose synthase genes (*CESA4*, *CESA7*, and *CESA9*) were screened and performed to correlation analysis with the three *PeMAP6*5s. The results indicated that the Pearson correlation coefficient between *MAP65-18* and SCW biosynthesis genes was 0.74–0.97, which was higher than those of *PeMAP65-1* and *PeMAP65-11* (Figure 8B). Among them, *PeMYB46*, as a homolog of the switch transcription factor *OsMYB46* contributing to the SCW biosynthesis in rice [28], has a high correlation coefficient of 0.92 with *PeMAP65-18*, potentially serving as a direct upstream transcription factor for *PeMAP65-18*. The result of qRT-PCR also confirmed that the expression pattern of *PeMYB46* across various internodes of moso bamboo shoots corresponded with the degree of lignification (Figure 8C). Two elements, M46R1 and M46R2, that could be bounded by MYB46 were discovered in the *PeMAP65-18* promoter (Figure 8D). The results of Y1H showed that PeMYB46 could bind to the *PeMAP65* promoter (Figure 8E). Furthermore, PeMYB46 could enhance the expression of *PeMAP65-18*, as verified by Dual-LUC assay (Figure 8F–H).

### 2.9. PeMAP65-18 Was Located in Microtubules

**Figure 9:**
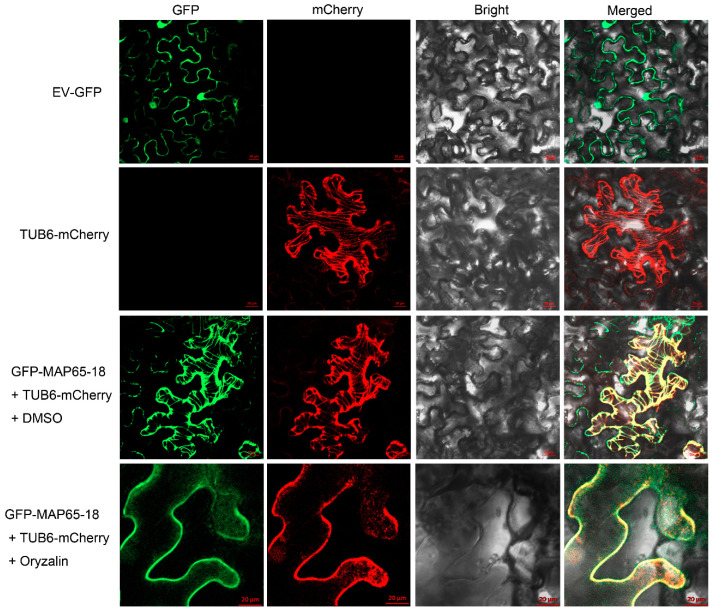
Subcellular localization of PeMAP65-18 in tobacco leaves. EV-GFP represents the empty vector containing *35S*::*GFP*. AtTUB6-mCherry used for microtubule localization marker. DMSO or oryzalin (100 μM) treatment for 30 min. Scale bars = 20 µm.

To detect the subcellular localization of PeMAP65-18, the *35S*::*eGFP-PeMAP65-18* vector was expressed in tobacco leaves. Through laser confocal microscopy observation, the fluorescence signal of eGFP-PeMAP65-18 showed a filamentous distribution, which was consistent with the signal of the microtubule localization marker AtTBU6-mCherry (Figure 9) and conformed to the distribution characteristics of cortical microtubules. Therefore, it was speculated that PeMAP65-18 was located in cortical microtubules. Further supporting this, the filamentous fluorescence signals of both eGFP-PeMAP65-18 and TUB6-mCherry were degraded following treatment with oryzalin, a microtubule depolymerizing agent, indicating that PeMAP65-18 is primarily associated with microtubules.

## 3. Discussion

### 3.1. Hybridization Events and Chromosome Doubling Drove the Expansion of the PeMAP65 Gene Family

In this study, nineteen MAP65s were identified in moso bamboo. Phylogenetic relationship analysis found that the four bamboo species and rice belonging to the Poaceae family had close genetic relationships. Collinear analysis in the genome of moso bamboo showed that there were 13 pairs of replication genes in the *PeMAP65* gene family, and all of them were segmented replications, which indicated that fragment replication was the main driving force for the evolution and expansion of the *PeMAP65* gene family. Herbaceous bamboos have the fewest homologous gene pairs with *P. edulis* due to their independent evolution. The divergence time between *P. edulis* and *R. guianensis* for *MAP65* genes was estimated to be as early as 34.40 million years ago, which aligns with the divergence of the four ancestral woody bamboo groups (ABCD) [29]. The whole-genome duplication in *P. edulis* occurred between 7 and 12 million years ago, indicating that the amplification of homologous genes between the genus *R. guianensis* and *P. edulis* occurred prior to this event. Around 19.40 million years ago, some tropical woody bamboos underwent two rounds of hybridization, resulting in a hexaploid genome (AABBCC), leading to the highest number of homologous gene pairs between *P. edulis* and *D. latiflorous*. The earliest divergence of *MAP65* genes between *P. edulis* and *D. latiflorous* occurred approximately 19.43 million years ago, which coincides with the formation of neotropical woody bamboo [30]. These results suggest that hybridization events among ancestral woody bamboos and chromosome doubling drove the expansion of the *PeMAP65* gene family.

### 3.2. Functional Differentiation Existed Among the Members of Different MAP65 Subfamilies

Microtubule-associated proteins, specifically MAP65s, play different roles in various biological processes. AtMAP65-1 functions in the early stages of cell division by interacting with microtubules in a sequential stepwise manner to form a fortress-like structure, thereby maintaining microtubule stability [31]. Conversely, AtMAP65-2 stabilizes cytoskeletal organization by promoting MT activity [32]. PeMAP65-13, PeMAP65-6, and PeMAP65-2 cluster with AtMAP65-1 and AtMAP65-2 in subfamily I, suggesting that these PeMAP65s may be broadly involved in mitosis and play critical roles in stabilizing microtubule structures. During mitosis, AtMAP65-4 is localized at the cortical division site and the central region of the spindle, where it regulates the formation of the spindle and kinetochore fibers [33]. The loss of *ATMAP65-3* function may result in the failure to initiate cytokinesis [34]. It was speculated that the members of the PeMAP65 subfamily V might play a critical role primarily in the late stages of mitosis, specifically during cytokinesis. A previous study has shown that AtMAP65-6 and AtMAP65-7 can associate with cortical microtubules [35]. PeMAP65-18 was clustered in subfamily VI and located in cortical microtubules, consistent with the localization of AtMAP65-6 and AtMAP65-7, indicating that they may have similar functions.

MAP65 is mainly located on microtubules in the cytoplasm and participates in the dynamic assembly and structural regulation of microtubule skeletons through specific binding to microtubules. In this study, 11 PeMAP65s were predicted to be in the cytoplasm, two in the chloroplast, and one in the endoplasmic reticulum. Although MAP65 is not directly associated with chloroplasts and endoplasmic reticulum, microtubules have been observed to interact with chloroplasts and endoplasmic reticulum in plants [36,37], and MAP65 proteins may be involved in modulating these interactions. However, five PeMAP65s were predicted to be in the nucleus. The results suggested that these PeMAP65s may be related to MT-organizing centers (MTOCs), which are situated on or near the nuclear envelope and which are derived from material related to the nuclear matrix. It may be generalized that some type of nucleus-related MT-organizing structure is basic to all mitotically dividing eukaryotic cells [38]. To fully understand the implications of these predictions, future studies should aim to experimentally validate the localization of these MAP65 proteins and investigate their potential roles in various cellular components.

The expression patterns of *PeMAP65*s were distinctly classified into two groups in shoots with different development stages. Cluster II primarily included members from subfamilies I and V, which were predominantly expressed in the early stage of shoot development, indicating that these *PeMAP65*s may be related to cell division in bamboo shoots. In contrast, cluster I contained most members of subfamily IV, which showed high expression in rapidly lignifying tissues. In the hierarchical regulatory network, the three identified *PeMAP65*s, all belonging to subfamily IV, were co-expressed with genes involved in cellulose and lignin biosynthesis and regulated by multiple transcription factors. These results suggest that *PeMAP65*s from subfamily IV may be involved in SCW formation by influencing the orientation of cortical MTs, particularly *PeMAP65-1*, *PeMAP65-11*, and *PeMAP65-18*.

### 3.3. PeMAP65-18 Regulated by PeMYB46 Has a Potential Role in SCW Formation

Current research has revealed the presence of a complex hierarchical transcriptional network within plants, mainly consisting of NAC and MYB transcription factors, which regulate the thickening of the SCW [39]. As secondary transcription factors, *MYB46* and *MYB83* directly activate downstream genes, including *MYB20*, *MYB42*, *MYB43*, and *MYB85*, as well as structural genes involved in cellulose and lignin biosynthesis [40,41]. Homologous genes of *AtMYB46*/*AtMYB83*, *PtrMYB3*, and *PtrMYB20* also participate in the regulation of SCW biosynthesis in poplar [42]. Similarly, overexpression of *OsMYB46* or *ZmMYB46* in *Arabidopsis* can directly induce SCW biosynthesis [28]. This multi-tiered, feed-forward transcriptional regulatory structure ensures precise control over SCW biosynthesis, and this regulatory network is highly conserved. In this study, the hierarchical regulatory network indicated that *PeMYB46*, a crucial transcription factor for SCW biosynthesis, may serve as an upstream transcription factor of *PeMAP65-18*. Y1H and Dual-LUC assays confirmed that *PeMYB46* could bind to the promoter and directly activate the transcription of *PeMAP65-18*. It is suggested that *PeMAP65-18* might play a crucial role in SCW formation of moso bamboo.

Cytoplasmic microtubules can be mainly divided into two subpopulations: cortical microtubules and perinuclear microtubules. Cortical microtubules are located beneath the plasma membrane and arranged parallel along its inner side, forming a network that maintains the cell shape, regulates the direction of cell growth, and plays a critical role in cell wall formation [38]. Perinuclear microtubules, on the other hand, surround the nucleus and other cytoplasmic structures, extending outward from the MTOC in a radial arrangement. These perinuclear microtubules are primarily responsible for intracellular material transport and play a vital role during cell division [38]. Cortical microtubules are essential for guiding the deposition of SCWs, as they regulate the shape, orientation, and pattern of cellulose microfibril synthesis. Early studies showed that cortical microtubules are aligned parallel to cellulose microfibrils, and disrupting these microtubules altered the orientation of the microfibrils, leading to the hypothesis that cortical microtubules mediate CSC movement [43]. Recent findings support this, showing that CSCs move along the trajectory of cortical microtubules [44]. Additionally, proteins like CELLULOSE SYNTHASE INTERACTING1/POM-POM2 (CSI1/POM2) act as bridges between the CSCs and microtubules during cellulose biosynthesis, further highlighting the role of cortical microtubules in SCW thickening [45]. Given the role of cortical microtubules in regulating SCW deposition and based on the distribution characteristics of PeMAP65-18-GFP fluorescence signals, it is speculated that PeMAP65-18 may be involved in bundling cortical microtubules and guiding cellulose microfibril synthesis during SCW formation. Previous studies have demonstrated that transient overexpression of *ZeMAP65-1* in *Arabidopsis* induced the formation of super-bundled microtubules, similar to patterns observed in xylem cells during differentiation [20]. These findings suggest that *PeMAP65-18* may similarly play a role in microtubule organization and cellulose synthesis. Future research will investigate the function of *PeMAP65*s in bamboo through genetic transformation to further elucidate their roles in SCW deposition.

## 4. Materials and Methods

### 4.1. Identification of PeMAP65 Members in Moso Bamboo

The genome sequence and annotation of moso bamboo were obtained from BambooGDB (http://bamboo.bamboogdb.org/, accessed on 21 April 2024). The PFAM database (http://pfam.xfam.org/, accessed on 21 April 2024) was utilized to identify proteins containing the MAP65 conservative domain (PF03999) in moso bamboo. The HMMER 3.0 program was then used to search for proteins with the MAP65 domain with a threshold of *E* < 1 × 10^−10^. The NCBI Conserved Domain Database (https://www.ncbi.nlm.nih.gov/Structure/cdd, accessed on 21 April 2024) was used to analyze the protein domain (*E* < 0.01). The molecular weight (MW) and isoelectric point (pI) of PeMAP65 proteins were obtained from ExPaSy (https://web.expasy.org/compute_pi/, accessed on 21 April 2024). The subcellular localization was provided by Plant-mPLoc (http://www.csbio.sjtu.edu.cn/bioinf/plant-multi, accessed on 21 April 2024).

### 4.2. Sequence Alignment and Phylogenetic Tree Construction

MAP65 proteins from six species (*Arabidopsis*, *O. sativa*, *P. trichocarpa*, *Dendrocalamus latiflorous*, *Guadua angustifolia*, *Raddia guianensis*) were aligned using Clustal W. Phylogenetic analysis was then performed using MEGA X (version 10.1.18) with the maximum likelihood method and bootstrap analysis with 1000 replicates. The phylogenetic tree was visually enhanced by Chiplot (https://www.chiplot.online/, accessed on 23 April 2024).

To compare MAP65 proteins in moso bamboo with those in other plant species (Arabidopsis, *O. sativa*, *P. trichocarpa*, *D. latiflorus*, *G. angustifolia*, *guianensis*), protein sequences were aligned using Clustal W. Phylogenetic analysis was conducted using MEGA X with the maximum likelihood method and 1000 bootstrap replicates, and the phylogenetic tree was refined using Evolview (https://evolgenius.info//evolview-v2, accessed on 23 April 2024).

### 4.3. Gene Structure, Chromosomal Location, and Motif Identification

The gene structure and chromosomal location of *PeMAP65*s were analyzed based on the genome annotation of moso bamboo. The Multiple EM for Motif Elicitation program (MEME) was employed to identify conserved motifs of PeMAP65 protein sequences (http://meme-suite.org/tools/meme, accessed on 25 April 2024). The results were visualized using TBtools-II (v2.119).

### 4.4. Prediction of Cis-Acting Regulatory Elements in PeMAP65 Promoters

The *cis*-acting elements of *PeMAP65*s promoter (2000 bp) were predicted by the PlantCARE online tool (http://bioinformatics.psb.ugent.be/webtools/plantcare/html/, accessed on 28 April 2024). The heatmap was generated using TBtools-II (v2.119).

### 4.5. Synteny and Gene Duplication Analysis

For analyzing scaffold distribution and syntenic relationships of PeMAP65s, the wyp1125/MCScanX software package was used. The *Ka* (non-synonymous substitution) to *Ks* (synonymous substitution) ratios were calculated. The syntelog analysis of *PeMAP65*s was performed according to the online database (https://bamboo.genobank.org/, accessed on 28 April 2024). The evolutionary time (T) was calculated using the *Ks* value with the formula: T = Ks/2λ, λ = 9.1 × 10^−9^ [46].

### 4.6. Expression Analysis of MAP65 Genes in Moso Bamboo

For the expression in different tissues and developmental stages, transcriptome data of 26 samples were obtained from the NCBI Sequence Read Archive (SRA) (accession number: SRX2408703-SRX2408728 and SRR13201212-SRR13201244) [47]. To explore the expression patterns in different internodes of bamboo shoots at various heights, transcriptome data were obtained from NCBI SRA with the BioProject IDs: PRJNA673565 and PRJNA682693.

### 4.7. qRT-PCR for PeMAP65s

Samples for qRT-PCR were collected in mid-April in Hangzhou, Zhejiang Province, China. Bamboo shoots measuring 2.5 m in height were chosen, and specific internodes were selected. The 22nd internode was labeled as S1, while the 18th internode was divided into two parts: upper and lower, labeled as S2 and S3. Similarly, the 13th internode was divided into three parts: upper, middle, and lower, labeled as S4, S5, and S6, respectively.

The total RNA of plant samples was extracted using the RNAprep Pure kit (Tiangen, Beijing, China). The PrimeScript 1st Strand cDNA Synthesis kit (Takara, Dalian, China) was employed to reverse transcribe to cDNA. The primers for quantitative real-time PCR (qRT-PCR) were designed using the Primer5 software (Table S1). The TB Green Premix Ex Taq^TM^ Kit (TaKaRa, Kusatsu, Japan) and QuantStudio^TM^7 Flex Real-time PCR instrument (Applied Biosystems, Carlsbad, CA, USA) were used. Results were calculated using the 2^−ΔΔCt^ method [48]. The *tonoplast intrinsic protein 41 (TIP41)* was used as a reference gene [49].

### 4.8. Construction of Co-Expression and Hierarchical Gene Regulatory Networks

The co-expression relationships between *PeMAP65s* and their edge genes were obtained through the BambooNET database (http://bioinformatics.cau.edu.cn/bamboo/, accessed on 23 April 2024) and visualized using Cytoscape software v3.10.2. The co-expressed genes were enriched by the GO and KEGG annotation.

From the 2097 genes exhibiting similar or opposing expression trends to *PeMAP65*s based on the transcriptomic data, 23 genes related to SCW biosynthesis were selected as the lowest tier of the regulatory network. Using the bottom-up GGM algorithm [50], a hierarchical regulatory map was generated encompassing *PeMAP65*s and its upstream genes using Cytoscape (v3.8.2).

### 4.9. Yeast Assays

One-hybrid yeast assays were conducted to determine the interaction between PeMYB46 and the promoter of *PeMAP65-18.* The recombined plasmid pB42AD-PeMYB46 and pLaczi-PeMAP65-18pro were co-transformed into yeast EYG48 strains. The transformed yeast cells were then grown on selective medium, specifically SD/-Trp-Ura, for three days at 30 °C. The positive colonies were selected and then plated on SD/-Trp/-Ura medium supplemented with X-gal (SD/-Trp/-Ura, 20% galactose, 20% raffinose, 20 mg/mL X-gal).

### 4.10. Dual-Luciferase Assays

The CDS of *PeMYB46* was cloned into the effector vector pGreenII 62-SK, while the promoter (2000 bp) of *PeMAP65-18* was cloned into the reporter vector pGreenII 0800-Luc and then transfected into leaves of 4-week-old tobacco. The activities of firefly luciferase (LUC) and renillia luciferase (REN) were measured using the Dual-Luciferase Reporter Assay System (Yeasen, Shanghai, China). Additionally, the fluorescence emitted from luciferase was observed using a low-light cooled CCD imaging apparatus (Lumazone, Singapore) after a Luciferin (100 μM) spray. The images were then processed and analyzed using Image J (Java 1.8.0_172).

### 4.11. Subcellular Locolization

The full-length coding sequence (CDS) of *PeMAP65-18* was cloned and then fused with the enhanced green fluorescent protein (eGFP) under the control of the *CaMV35S* promoter. The recombinant vectors containing the eGFP-PeMAP65 fusion construct were transformed into *Agrobacterium tumefaciens* strain GV3101 and infected the tobacco leaves. *β*-tubulin (TUB6) of *Arabidopsis* was fused with mCherry as a tubulin marker. After 36 h of infection, tobacco leaves were treated with DMSO or oryzalin for 30 min, respectively. Fluorescence signals were observed on a laser confocal microscope from ZEISS (model LSM 880).

### 4.12. Data Collection and Statistical Analysis

The figures delineated in this research are conveyed as means ± standard deviation (SD), derived from no less than three independent trials. Statistical significance of the outcomes was ascertained via one-way analysis of variance (ANOVA). GraphPad Prism 12.0 software was utilized for the execution of these statistical examinations.

## 5. Conclusions

In this study, 19 MAP65s were identified from moso bamboo, and a phylogenetic tree of MAP65s from multiple species was constructed, dividing them into five subgroups. The PeMAP65s within the same subgroup shared similar gene structures and conserved domains. Segmental duplication appeared to be the primary driving force behind the expansion of the *MAP65* gene family, which has been subject to purifying selection pressure during evolution. Additionally, chromosome doubling and hybridization events between woody bamboos contributed to the expansion of the *PeMAP65s* in moso bamboo. The functional differentiation was investigated in the *PeMAP65*s based on the evolutionary relationship and expression patterns, which could be categorized into two clusters: one related to cell division and the other involved in SCW formation. The expression of *PeMAP65-18* was positively correlated with the expression of genes related to SCW biosynthesis and activated by PeMYB46, a pivotal transcription factor involved in SCW formation. Subcellular localization has confirmed that MAP65-18 is located in microtubules, and it was speculated that PeMAP65-18 was responsible for bundling cortical microtubules and guiding cellulose microfibril synthesis during SCW deposition. In summary, the comprehensive analysis of the PeMAP65 family offers novel insights into the roles of MAP65s in moso bamboo, particularly regarding SCW formation.

## Figures and Tables

**Table 1:** Basic characteristic of PeMAP65s.

Gene Name	Gene ID	ORF (aa)	MW (kDa)	pI	Sub Localization
*PeMAP65-1*	*PH02Gene22090.t1*	595	67.26	7.56	nucleus
*PeMAP65-2*	*PH02Gene30151.t3*	593	66.28	5.08	chloroplast
*PeMAP65-3*	*PH02Gene42885.t1*	583	65.59	5.34	cytoplasm
*PeMAP65-4*	*PH02Gene22734.t4*	676	76.84	5.74	cytoplasm
*PeMAP65-5*	*PH02Gene01427.t1*	617	70.27	6.41	cytoplasm
*PeMAP65-6*	*PH02Gene18145.t1*	582	65.06	5.07	cytoplasm
*PeMAP65-7*	*PH02Gene25608.t1*	577	65.03	5.52	cytoplasm
*PeMAP65-8*	*PH02Gene18421.t1*	561	63.74	6.26	cytoplasm
*PeMAP65-9*	*PH02Gene25799.t1*	590	68.31	6.97	cytoplasm
*PeMAP65-10*	*PH02Gene20031.t1*	687	78.07	5.83	cytoplasm
*PeMAP65-11*	*PH02Gene19121.t2*	610	69.57	6.27	nucleus
*PeMAP65-12*	*PH02Gene25122.t1*	614	68.5	8.2	nucleus
*PeMAP65-13*	*PH02Gene27598.t2*	510	58.7	5.33	endoplasmic reticulum
*PeMAP65-14*	*PH02Gene14605.t1*	636	71.55	5.35	nucleus
*PeMAP65-15*	*PH02Gene39808.t1*	591	67	6.78	nucleus
*PeMAP65-16*	*PH02Gene10237.t1*	584	66.63	5.15	cytoplasm
*PeMAP65-17*	*PH02Gene34993.t1*	641	73.39	6.41	chloroplast
*PeMAP65-18*	*PH02Gene23444.t3*	577	65.09	7.9	cytoplasm
*PeMAP65-19*	*PH02Gene45477.t1*	591	68.42	6.4	cytoplasm

## Data Availability

Data are contained within the article and Supplementary Materials.

## Supplementary Materials

The following supporting information can be downloaded at: https://www.mdpi.com/article/10.3390/plants13213000/s1, Table S1: Primer sequences for qRT-PCR. Table S2: *Ks* and *Ka* analysis of duplicated gene pairs.
