# Supplementary material for: The Potential Role of PeMAP65-18 in Secondary Cell Wall Formation in Moso Bamboo
Source: Plants (Basel). 2024 Oct 27;13(21):3000. doi: 10.3390/plants13213000 (PMC11548304; doi:10.3390/plants13213000)
Supplement: Supplementary file 1 [file plants-13-03000-s001.zip › plants-3249858-supplementary.pdf]

Table S1 Primer sequences for qRT-PCR

Gene name	Gene ID	5' primers	3' primers
PeMAP65-1	*PH02Gene22090.t1*	CTAAACCAAGCCCAAAGAGGAC	AGTACCACTGCCAAGGGATGAT
PeMAP65-2	*PH02Gene30151.t3*	GACATCTATGCTGGTGCTCACAT	CACGCAGACATCCATCTTTCA
PeMAP65-3	*PH02Gene42885.t1*	AACTTGCCAACTCCAAATCCC	CAAACTCTTTCGCCCTCTTCTC
PeMAP65-4	*PH02Gene22734.t4*	CCAATACACCGATGCCACAA	GAGAATCCGCCGCTCATAAT
PeMAP65-5	*PH02Gene01427.t1*	TCAATGCAACCTCCAAAATCAG	TCGGCAATGTCCAAACCTCTA
PeMAP65-6	*PH02Gene18145.t1*	ACTGTATTGGGGATGGATTTTCTC	CTTCATTAAGCGTGGCTACTGTCT
PeMAP65-7	*PH02Gene25608.t1*	CCTTCAACAACTTGCCAACTCC	CATTAGCAAACTCTTCCACCCTCT
PeMAP65-8	*PH02Gene18421.t1*	TTGTGCATAAGAGGGCTGAGAA	AAAGTAGAACGCCATCGTAAGTGA
PeMAP65-9	*PH02Gene25799.t1*	TGAAGATGATATGGGATGAAGTCG	AAGGGAAAGAAGCAGATTGGTGA
PeMAP65-10	*PH02Gene20031.t1*	CGAGAATAGGACCCCAAAGACA	AAACAAGGGAAACGGGAGTAAC
PeMAP65-11	*PH02Gene19121.t2*	GACGAGCAGTCATCATCCAAAT	GCCTGTTCTCCTTGAAGTAGTTATTGT
PeMAP65-12	*PH02Gene25122.t1*	CAGATAGCGAAAGCCAAGGAA	CCAGTATTGAATCTGTTCTCGTCC
PeMAP65-13	*PH02Gene27598.t2*	TACAACCGAAACGAGAACAGATACA	CGAGGAAACAAGAAAAGACATACACC
PeMAP65-14	*PH02Gene14605.t1*	ACACCAGGTAGAAGACTATCAATCACA	TGTCGCTGCTTCATTCAGGTAA
PeMAP65-15	*PH02Gene39808.t1*	CTGGCAAATATCGAGTCACAAATAC	TCTTCATCACAAGCAGCAATCC
PeMAP65-16	*PH02Gene10237.t1*	CCACTTCGTGAACTCACTCCTACT	ATTGCCCGAGTCAAAGATGTAA
PeMAP65-17	*PH02Gene34993.t1*	ATAGCAACCATAAAGAAGACGGAG	CACAAGACCAGAATCAATCAACG
PeMAP65-18	*PH02Gene23444.t3*	TATTCTGGGCGGTACAATAACTACT	CTAATGGAGGCAAAGGATGACA
PeMAP65-19	*PH02Gene45477.t1*	ATGGGATCACTCAGGATGACTGGAA	TTCCAGTCATCCTGAGTGATCCCAT

Table S2 Ks and Ka analysis of duplicated gene pairs

Gene name	Gene ID	Ka	Ks	Ka/Ks	Duplication time/（Million years ago）
PeMAP65-6	*PeMAP65-11*	0.1155	0.9360	0.1234	51.4268
PeMAP65-6	*PeMAP65-9*	0.0410	0.1386	0.2956	7.6170
PeMAP65-9	*PeMAP65-11*	0.1159	0.9242	0.1254	50.7778
PeMAP65-7	*PeMAP65-3*	0.0210	0.1270	0.1653	6.9794
PeMAP65-14	*PeMAP65-3*	0.1441	0.6078	0.2371	33.3958
PeMAP65-8	*PeMAP65-4*	0.0356	0.1384	0.2574	7.6063
PeMAP65-7	*PeMAP65-14*	0.1401	0.6370	0.2199	34.9974
PeMAP65-2	*PeMAP65-16*	0.0303	0.1133	0.2677	6.2244
PeMAP65-12	*PeMAP65-19*	0.0242	0.1066	0.2271	5.8564
PeMAP65-17	*PeMAP65-15*	0.0927	0.2313	0.4007	12.7069
PeMAP65-10	*PeMAP65-20*	0.0270	0.1115	0.2424	6.1279
PeMAP65-13	*PeMAP65-18*	0.0353	0.1521	0.2319	8.3569
PeMAP65-6	*PeMAP65-11*	0.1155	0.9360	0.1234	51.4268
PeMAP65-6	*PeMAP65-9*	0.0410	0.1386	0.2956	7.6170
PeMAP65-9	*PeMAP65-11*	0.1159	0.9242	0.1254	50.7778
PeMAP65-7	*PeMAP65-3*	0.0210	0.1270	0.1653	6.9794
PeMAP65-14	*PeMAP65-3*	0.1441	0.6078	0.2371	33.3958
PeMAP65-8	*PeMAP65-4*	0.0356	0.1384	0.2574	7.6063
PeMAP65-7	*PeMAP65-14*	0.1401	0.6370	0.2199	34.9974
PeMAP65-9	*RgMAP65-8*	0.0485	0.2423	0.2000	13.3154
PeMAP65-10	*RgMAP65-1*	0.0399	0.3089	0.1292	16.9749
PeMAP65-11	*RgMAP65-3*	0.0343	0.2515	0.1363	13.8176
PeMAP65-12	*RgMAP65-4*	0.0777	0.3283	0.2367	18.0377
PeMAP65-13	*RgMAP65-2*	0.0773	0.2955	0.2617	16.2351
PeMAP65-15	*RgMAP65-10*	0.0459	0.3134	0.1464	17.2201
PeMAP65-16	*RgMAP65-9*	0.1361	0.6261	0.2174	34.4001
PeMAP65-17	*RgMAP65-4*	0.0780	0.3362	0.2321	18.4701
PeMAP65-19	*RgMAP65-8*	0.0477	0.2861	0.1667	15.7183
PeMAP65-1	*RgMAP65-10*	0.0518	0.2760	0.1875	15.1624
PeMAP65-3	*RgMAP65-6*	0.0502	0.2954	0.1700	16.2319
PeMAP65-2	*RgMAP65-7*	0.0217	0.2662	0.0817	14.6245
PeMAP65-4	*RgMAP65-5*	0.0518	0.2893	0.1790	15.8969
PeMAP65-6	*RgMAP65-7*	0.0260	0.2602	0.0997	14.2973
PeMAP65-7	*RgMAP65-6*	0.0672	0.3154	0.2129	17.3306
PeMAP65-9	*GanMAP65-14*	0.0565	0.2047	0.2761	11.2445
PeMAP65-9	*GanMAP65-15*	0.0340	0.0927	0.3673	5.0928
PeMAP65-10	*GanMAP65-1*	0.1184	0.2869	0.4126	15.7652
PeMAP65-10	*GanMAP65-2*	0.0262	0.1359	0.1926	7.4662

PeMAP65-11	*GanMAP65-5*	0.0403	0.1816	0.2219	9.9783
PeMAP65-12	*GanMAP65-6*	0.0309	0.1438	0.2146	7.9011
PeMAP65-12	*GanMAP65-7*	0.0502	0.1338	0.3753	7.3540
PeMAP65-11	*GanMAP65-12*	0.0326	0.0941	0.3467	5.1692
PeMAP65-14	*GanMAP65-3*	0.1043	0.3005	0.3470	16.5132
PeMAP65-13	*GanMAP65-4*	0.0910	0.2224	0.4092	12.2175
PeMAP65-15	*GanMAP65-16*	0.0305	0.2003	0.1521	11.0073
PeMAP65-15	*GanMAP65-18*	0.0793	0.1769	0.4482	9.7209
PeMAP65-16	*GanMAP65-3*	0.1576	0.4025	0.3917	22.1132
PeMAP65-17	*GanMAP65-6*	0.0437	0.1526	0.2865	8.3864
PeMAP65-17	*GanMAP65-7*	0.0638	0.1931	0.3306	10.6083
PeMAP65-19	*GanMAP65-14*	0.0546	0.2420	0.2257	13.2993
PeMAP65-19	*GanMAP65-15*	0.0318	0.1195	0.2661	6.5646
PeMAP65-1	*GanMAP65-16*	0.0338	0.1783	0.1896	9.7986
PeMAP65-1	*GanMAP65-18*	0.0857	0.2346	0.3651	12.8901
PeMAP65-3	*GanMAP65-13*	0.0315	0.1008	0.3128	5.5380
PeMAP65-4	*GanMAP65-9*	0.0528	0.1716	0.3077	9.4259
PeMAP65-5	*GanMAP65-8*	0.0494	0.1696	0.2912	9.3211
PeMAP65-4	*GanMAP65-10*	0.0372	0.1414	0.2634	7.7698
PeMAP65-7	*GanMAP65-13*	0.0433	0.1506	0.2878	8.2746
PeMAP65-8	*GanMAP65-8*	0.0677	0.1832	0.3692	10.0677
PeMAP65-10	*DlMAP65-2*	0.0321	0.1191	0.2698	6.5457
PeMAP65-10	*DlMAP65-1*	0.0315	0.1243	0.2534	6.8275
PeMAP65-4	*DlMAP65-22*	0.0323	0.1195	0.2707	6.5651
PeMAP65-4	*DlMAP65-23*	0.0331	0.1169	0.2831	6.4234
PeMAP65-5	*DlMAP65-25*	0.0393	0.1614	0.2438	8.8666
PeMAP65-8	*DlMAP65-24*	0.1373	0.2944	0.4665	16.1740
PeMAP65-4	*DlMAP65-26*	0.0290	0.0974	0.2977	5.3493
PeMAP65-4	*DlMAP65-27*	0.0282	0.1004	0.2807	5.5184
PeMAP65-2	*DlMAP65-29*	0.0236	0.1222	0.1933	6.7124
PeMAP65-3	*DlMAP65-31*	0.0353	0.1245	0.2832	6.8432
PeMAP65-3	*DlMAP65-28*	0.0320	0.1222	0.2620	6.7117
PeMAP65-7	*DlMAP65-28*	0.0421	0.1658	0.2537	9.1109
PeMAP65-7	*DlMAP65-31*	0.0453	0.1614	0.2806	8.8703
PeMAP65-6	*DlMAP65-29*	0.0267	0.1323	0.2020	7.2719
PeMAP65-2	*DlMAP65-32*	0.0217	0.1024	0.2123	5.6260
PeMAP65-2	*DlMAP65-33*	0.0164	0.1183	0.1390	6.4983
PeMAP65-6	*DlMAP65-32*	0.0244	0.1311	0.1865	7.2031
PeMAP65-6	*DlMAP65-33*	0.0244	0.1499	0.1629	8.2384
PeMAP65-12	*DlMAP65-39*	0.0221	0.0727	0.3038	3.9932
PeMAP65-17	*DlMAP65-39*	0.0388	0.1569	0.2475	8.6191

PeMAP65-2	*DlMAP65-34*	0.0202	0.1115	0.1813	6.1286
PeMAP65-2	*DlMAP65-36*	0.0205	0.1193	0.1716	6.5524
PeMAP65-6	*DlMAP65-34*	0.0221	0.1687	0.1312	9.2697
PeMAP65-6	*DlMAP65-36*	0.0199	0.1751	0.1134	9.6229
PeMAP65-10	*DlMAP65-4*	0.0403	0.1532	0.2630	8.4198
PeMAP65-10	*DlMAP65-3*	0.0396	0.1479	0.2680	8.1288
PeMAP65-9	*DlMAP65-41*	0.0257	0.0751	0.3416	4.1262
PeMAP65-9	*DlMAP65-42*	0.0256	0.0722	0.3553	3.9667
PeMAP65-19	*DlMAP65-41*	0.0263	0.1017	0.2583	5.5883
PeMAP65-19	*DlMAP65-42*	0.0263	0.1018	0.2581	5.5909
PeMAP65-15	*DlMAP65-44*	0.0295	0.1717	0.1718	9.4337
PeMAP65-15	*DlMAP65-43*	0.0302	0.1786	0.1694	9.8106
PeMAP65-1	*DlMAP65-44*	0.0358	0.1500	0.2388	8.2424
PeMAP65-1	*DlMAP65-43*	0.0366	0.1567	0.2335	8.6075
PeMAP65-15	*DlMAP65-45*	0.0277	0.1508	0.1834	8.2846
PeMAP65-15	*DlMAP65-46*	0.0288	0.1556	0.1849	8.5486
PeMAP65-1	*DlMAP65-46*	0.0306	0.1378	0.2221	7.5715
PeMAP65-1	*DlMAP65-45*	0.0291	0.1406	0.2070	7.7258
PeMAP65-10	*DlMAP65-5*	0.0297	0.0926	0.3210	5.0860
PeMAP65-10	*DlMAP65-6*	0.0317	0.0852	0.3721	4.6799
PeMAP65-13	*DlMAP65-7*	0.0390	0.1211	0.3223	6.6541
PeMAP65-13	*DlMAP65-10*	0.0390	0.1271	0.3071	6.9831
PeMAP65-16	*DlMAP65-8*	0.1187	0.3536	0.3357	19.4273
PeMAP65-11	*DlMAP65-14*	0.0341	0.0861	0.3961	4.7314
PeMAP65-11	*DlMAP65-15*	0.0374	0.0930	0.4025	5.1109
PeMAP65-12	*DlMAP65-16*	0.0354	0.1492	0.2374	8.1966
PeMAP65-17	*DlMAP65-16*	0.0428	0.1506	0.2845	8.2750
PeMAP65-11	*DlMAP65-18*	0.0278	0.0756	0.3681	4.1557
PeMAP65-11	*DlMAP65-19*	0.0282	0.0771	0.3655	4.2386
PeMAP65-3	*DlMAP65-17*	0.0286	0.0801	0.3568	4.4037
PeMAP65-3	*DlMAP65-20*	0.0269	0.0878	0.3066	4.8263
PeMAP65-7	*DlMAP65-17*	0.0427	0.1224	0.3488	6.7275
PeMAP65-7	*DlMAP65-20*	0.0403	0.1223	0.3300	6.7182
